# Glucose-6-Phosphate Dehydrogenase Deficiency in Nigerian Children

**DOI:** 10.1371/journal.pone.0068800

**Published:** 2013-07-12

**Authors:** Olatundun Williams, Daniel Gbadero, Grace Edowhorhu, Ann Brearley, Tina Slusher, Troy C. Lund

**Affiliations:** 1 Department of Pediatrics, University of Minnesota, Minneapolis, Minnesota, United States of America; 2 Division of Global Health, University of Minnesota, Minneapolis, Minnesota, United States of America; 3 Department of Pediatrics, Bowen University Teaching Hospital, Ogbomoso, Nigeria; 4 Department of Laboratory Medicine, Bowen University Teaching Hospital, Ogbomoso, Nigeria; 5 Biostatistical Design and Analysis Center, Clinical and Translational Science Institute, University of Minnesota, Minneapolis, Minnesota, United States of America; 6 Division of Blood and Marrow Transplant, University of Minnesota, Minneapolis, Minnesota, United States of America; Instituto de Higiene e Medicina Tropical, Portugal

## Abstract

Glucose-6-phosphate dehydrogenase (G6PD) deficiency is the most common human enzymopathy and in Sub-Saharan Africa, is a significant cause of infection- and drug-induced hemolysis and neonatal jaundice. Our goals were to determine the prevalence of G6PD deficiency among Nigerian children of different ethnic backgrounds and to identify predictors of G6PD deficiency by analyzing vital signs and hematocrit and by asking screening questions about symptoms of hemolysis. We studied 1,122 children (561 males and 561 females) aged 1 month to 15 years. The mean age was 7.4±3.2 years. Children of Yoruba ethnicity made up the largest group (77.5%) followed by those Igbo descent (10.6%) and those of Igede (10.2%) and Tiv (1.8%) ethnicity. G6PD status was determined using the fluorescent spot method. We found that the overall prevalence of G6PD deficiency was 15.3% (24.1% in males, 6.6% in females). Yoruba children had a higher prevalence (16.9%) than Igede (10.5%), Igbo (10.1%) and Tiv (5.0%) children. The odds of G6PD deficiency were 0.38 times as high in Igbo children compared to Yoruba children (p = 0.0500). The odds for Igede and Tiv children were not significantly different from Yoruba children (p = 0.7528 and 0.9789 respectively). Mean oxygen saturation, heart rate and hematocrit were not significantly different in G6PD deficient and G6PD sufficient children. The odds of being G6PD deficient were 2.1 times higher in children with scleral icterus than those without (p = 0.0351). In conclusion, we determined the prevalence of G6PD deficiency in Nigerian sub-populations. The odds of G6PD deficiency were decreased in Igbo children compared to Yoruba children. There was no association between vital parameters or hematocrit and G6PD deficiency. We found that a history of scleral icterus may increase the odds of G6PD deficiency, but we did not exclude other common causes of icterus such as sickle cell disease or malarial infection.

## Introduction

Glucose-6-phosphate dehydrogenase (G6PD) deficiency affects some 400 million people worldwide and is the most common human enzymopathy [1]. G6PD is found in the cytoplasm of all cells and is responsible for the production of the reduced form of nicotinamide adenine dinucleotide phosphate (NADPH), a coenzyme which maintains the intracellular pool of reduced glutathione and thereby protects cells against oxidative damage. Deficiency of G6PD particularly impacts red blood cells, as they lack nuclei and mitochondria and must therefore rely solely on G6PD for production of NADPH and reduced glutathione and protection from oxidative challenge.

In regions of the world such as tropical Africa the prevalence of G6PD deficiency ranges from 15–26%^2^. The public health burden of this condition is significant. G6PD deficiency contributes to neonatal jaundice which is accompanied by hyperbilirubinemia and puts infants at risk for kernicterus within the first few days of life. Kernicterus can lead to hearing deficits, behavior problems, and permanent neurologic damage. During childhood, many children with G6PD deficiency are healthy until they are exposed to a pro-oxidant medication or chemical. Classically, anti-malarial drugs are strong pro-oxidants and have substantial use in Sub-Saharan Africa. Additionally, exposure to the pro-oxidant naphthalene, the active ingredient in mothballs, is common among young children. In G6PD deficient children, pro-oxidant exposure can lead to a rapid imbalance in the redox status in red blood cells leading to hemolysis and resultant severe anemia, heart failure, and even death if not recognized early.

The WHO recommends population screening in regions were the prevalence of G6PD deficiency is 3–5% or more [2], but this has yet to become routine practice in many parts of Nigeria. Barriers to screening include cost, underestimation of the public health impact of G6PD deficiency by the medical community, lack of awareness of G6PD deficiency among lay people and a paucity of guidelines regarding which high risk groups should be preferentially screened when general population screening is not possible. There are several screening methods available, one of which is the fluorescent spot test^3^.

In this study, we employed the fluorescent spot test to determine the prevalence of G6PD deficiency in the Southwestern Nigerian town of Ogbomoso and some of its neighboring villages. The estimated population of Ogbomoso is just over one million. There are two major hospitals in the town, neither of which routinely screen children for G6PD deficiency. In the course of this study, we also investigated whether simple measures such as analysis of heart rate and oxygen saturation or screening questions about symptoms of hemolysis could identify healthy children with G6PD deficiency.

## Methods

### Study Sites and Sample Recruitment

This study took place between November and December 2011 in the town of Ogbomoso and the neighboring villages of Adodo, Ajinapa and Ilota in Southwestern Nigeria. The majority of Ogbomoso’s inhabitants are of Yoruba ethnicity. There is also a small community of people of Igbo descent in the town. The small villages of Adodo, Ajinapa and Ilota are home to the Tiv and Igede, people who have emigrated to the region from the central eastern state of Benue.

**Figure 1:**
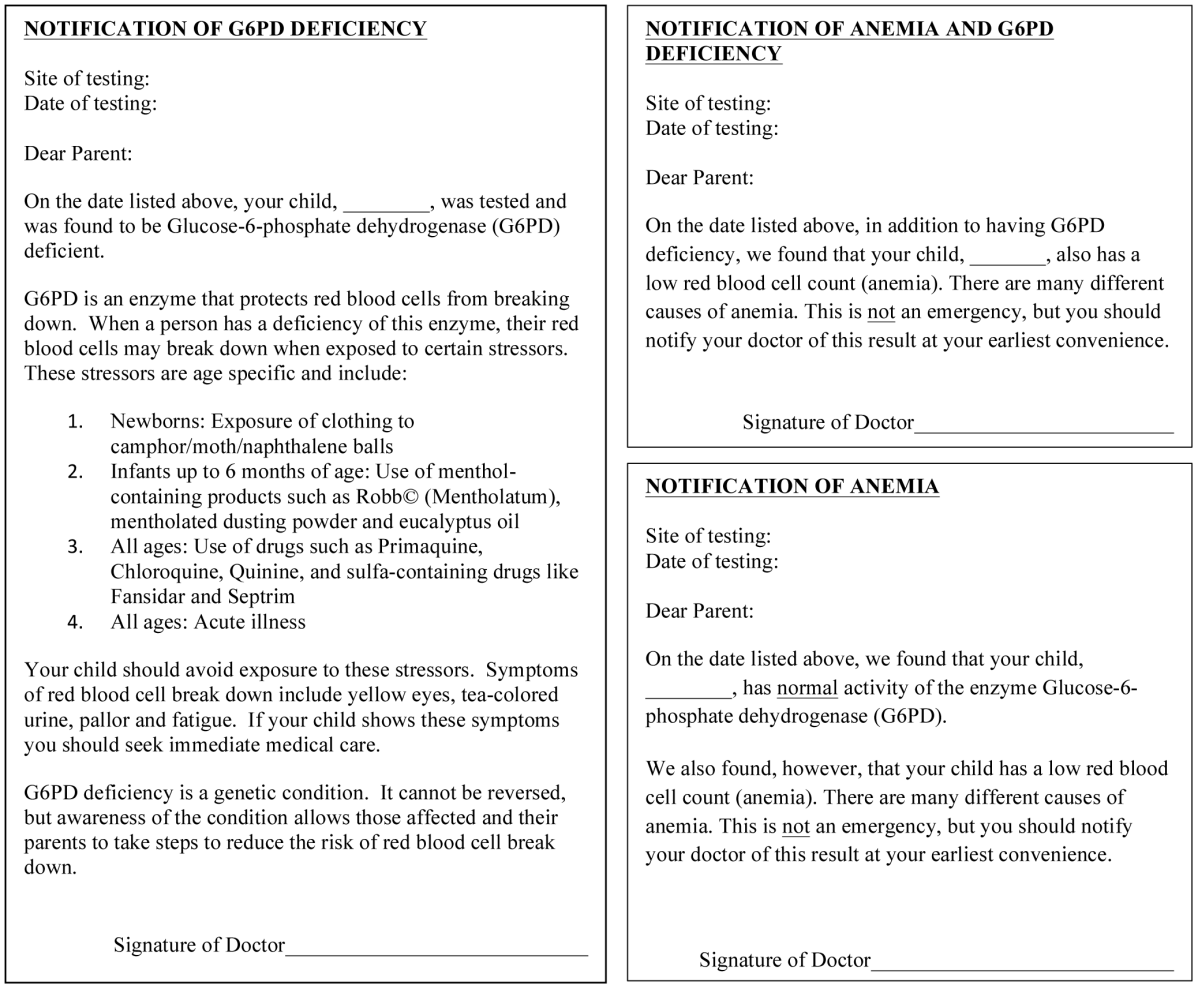
Result notification forms.

In order to obtain a representative sample of children from the various ethnic groups we traveled to their home villages and visited locales such as schools and places of worship where we were likely to find children of all ages and where applicable, those of varying socio-economic background. At these sites, parents were informed of the study and, if interested, were asked to sign a written consent to allow their children to participate. Translators were used for consent and recruitment in the local language. Children aged 15 years old or younger were invited to participate. Siblings within a family were included in the study. No known ill children were included in the analysis. Participants and their families did not receive compensation, however, children were given a small token (a sticker, a pen or a single serving packet of biscuits) after they had their blood collected. As supplies permitted, children who did not participate in the study also received one of the aforementioned small tokens. Parents of study participants were notified of their children’s test results using one of the following notification forms (Figure 1). This study and the use of all bodily fluids were approved by the Committee on the Use of Human Subjects in Research at the University of Minnesota and the Bowen University Teaching Hospital, Ogbomoso, Nigeria. Informed written consent was obtained for all patient samples from the parents or guardians on behalf of the child participants. Patient written assent was also obtained if patients were greater than 8 years of age.

### Screening and Sample Collection

Screening took place at schools and churches and consisted of documenting oxygen saturation and heart rate using a portable oximeter, and asking several simple screening questions of children aged 8 years or older or those with a parent present (Table 1). We limited screening questions to those that involved visible signs of hemolysis, as these were signs that we felt a child of at least 8 years of age would be likely to remember and accurately report. A few drops of blood were obtained by finger stick for G6PD assay and hematocrit determination.

**Table 1:** Screening questions about symptoms of hemolysis.

Question 1: Was your child jaundiced in the neonatal period?
Question 2: Have you ever had yellow eyes? Has your child? (if parent-directed)
Question 3: Have you ever had tea-colored urine? Has your child? (if parent-directed)

### G6PD Status Determination

Blood samples were collected in heparinized Microtainer® tubes and stored at room temperature. Samples were analyzed within 24 hours of collection (and generally on the same day). G6PD status was determined using the fluorescent spot method developed by Beutler and Mitchell[3]. The fluorescent spot test works on the principle that NADPH, which is produced from NADP in a reaction catalyzed by G6PD, fluoresces under long-wave ultraviolet light. A fluorescent spot indicates G6PD activity is present.

### Hematocrit Determination

For the majority of the samples (95%), the hematocrit was determined by capillary tube method. Capillary tubes were spun in a hematocrit centrifuge and then analyzed using the Hematastat II® machine. For samples that did not have an adequate amount of blood for capillary tube hematocrit determination, the HemoCue® Hb 201+ Analyzer was used.

### Data Analysis

**Figure 2:**
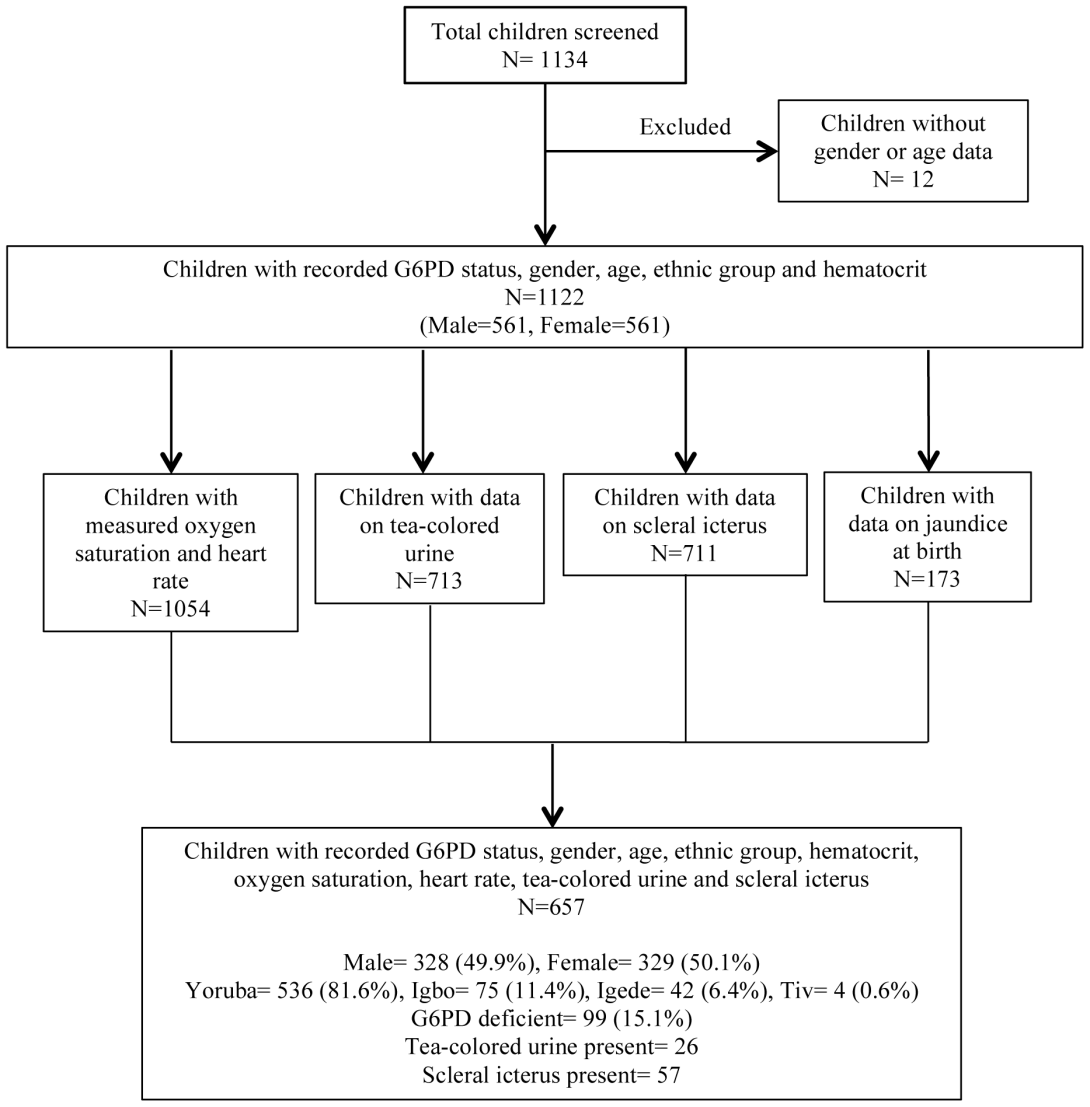
Data obtained from sampled population.

Data analysis was based on the total number of children with complete data for each predictor of interest (Figure 2). Statistical analysis was carried out using SAS 9.2.

Univariate tests of the association of each predictor with G6PD deficiency were carried out using the Pearson’s chi-square test or Fisher’s exact test for categorical predictors and the Satterthwaite *t*-test for continuous predictors.

In order to check for a correlation between age and hematocrit that could interfere with multivariate regression, hematocrit was plotted against both raw age and ordinal age. A linear model best predicted the correlation between hematocrit and age with a significant positive effect of age (t-test on regression slope, p<0.0001). The correlation, however, was not very high (0.313 for raw age and 0.314 for ordinal age), suggesting that we could confidently use both variables in a multivariate model without concern for collinearity.

Multivariate analysis was carried out on the 657 children who had complete data both on the outcome (G6PD status) and eight predictors of interest: gender, age, ethnic group, hematocrit, oxygen saturation, heart rate, tea-colored urine and scleral icterus. Information on jaundice at birth was available for only 173 of 1122 children (15%), so it was not included as a predictor.

A generalized linear mixed model for the log odds of being G6PD deficient, with the eight predictors of interest as fixed effects and a random effect of family, was fit to the data. The model was then refit without the random effect. For this dataset, the fit of the model was not significantly different without the random effect of family (likelihood ratio test, p = 0.2059), indicating that it was not necessary to account for within-family correlation in the model. After removal of the random effect, backward selection was used to reduce the number of predictors in the model. At each step, the least significant remaining predictor was selected and the fit of the models with and without that predictor were compared using likelihood ratio tests. If the fits were not significantly different, then this predictor was kept out. This process was continued until removing a predictor made the model fit significantly worse or until all the remaining predictors were significant. Heart rate, hematocrit, age, tea-colored urine, oxygen saturation, scleral icterus and ethnic group were sequentially tested with no significant change in the fit of the models (likelihood ratio test p-values 0.5839, 0.4795, 0.4028, 0.3173, 0.2367, 0.0652 and 0.1116 respectively) or the fitted odds ratios, and so the results for the full model are reported here.

Univariate models without random effect were also fit to the data for comparison.

## Results

A total of 1,122 children (561 males and 561 females) were screened for G6PD deficiency. The majority of children were of Yoruba ethnicity (77.5%). Children of Igbo decent made up the second largest group (10.6%), followed by those of Igede (10.2%) and Tiv (1.8%) ethnicity. Participants ranged in age from 1 month to 15 years. The mean age was 7.4±3.2 years. Children of age 6 years or younger made up 35.6% of participants. A total of 448 children belonged to 177 distinct families. These families contained 2 to 6 children. The characteristics of the population are summarized in Table 2.

**Table 2:** Population characteristics.

		Yoruba	Igbo	Tiv/Igede	Total
**Gender**	**Female**	433 (49.8%)	56 (47.1%)	72 (53.7%)	561 (50%)
	**Male**	436 (50.2%)	63 (52.9%)	62 (46.3%)	561 (50%)
**Age**	**≤6 months**	6 (0.7%)	5 (4.2%)	9 (6.7%)	20 (1.8%)
	**7 months- 2 years**	38 (4.4%)	26 (21.8%)	25 (18.7%)	89 (7.9%)
	**3–6 years**	208 (23.9%)	33 (27.7%)	49 (36.6%)	290 (25.8%)
	**7–10 years**	484 (55.7%)	35 (29.4%)	33 (24.6%)	552 (49.2%)
	**11–15 years**	133 (15.3%)	20 (16.8%)	18 (13.4%)	171 (15.2%)

The overall prevalence of G6PD deficiency was 15.3% in all children. In males, the prevalence was 24.1% and in females it was 6.6%. In our multivariate analysis, the odds of being G6PD deficient were 3.6 times higher in males than in females (p<0.0001).

**Figure 3:**
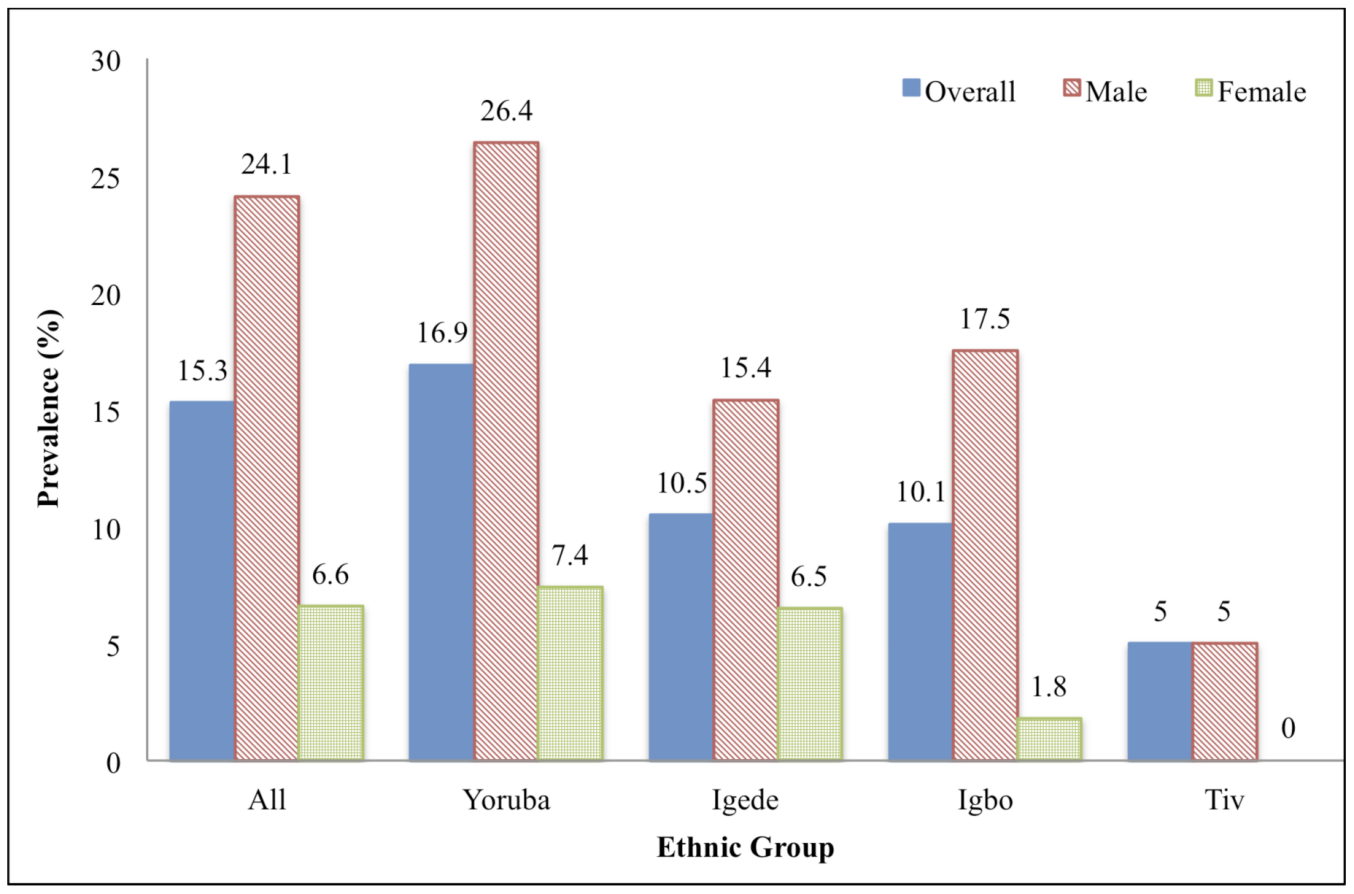
Prevalence of Glucose-6-phosphate dehydrogenase deficiency amongst Nigerian ethnic groups. Multivariate analysis indicated that ethnic group was associated with G6PD deficiency: the odds of being deficient were 0.38 times as high in Igbo children compared to Yoruba children (p = 0.0500). The odds for Igede and Tiv children were not significantly different from Yoruba children (p = 0.7528 and 0.9789 respectively).

The prevalence of G6PD deficiency varied between ethnic groups with Yoruba children having a higher prevalence (16.9%) than Igede (10.5%), Igbo (10.1%) and Tiv (5.0%) children as shown in Figure 3. Based on our multivariate analysis, ethnic group may be associated with G6PD deficiency with the odds of deficiency being 0.38 times as high in Igbo children compared to Yoruba children (Table 3, p = 0.0500), although the association was not significant after accounting for multiple testing. The odds for Igede and Tiv children were not significantly different from Yoruba children (p = 0.7528 and 0.9789 respectively). When we looked at the gender-specific rates of G6PD deficiency, the rate of deficiency did not vary significantly across ethnic groups in either females or males (p = 0.3531 and 0.1114 respectively).

**Table 3:** Relationship between G6PD deficiency and gender, age, ethnic group, hematocrit, oxygen saturation, heart rate, tea-colored urine and scleral icterus.

N = 657	Univariate Models:	Multivariate Model:	
	Unadjusted Odds Ratio	Adjusted Odd Ratio	
Predictor	Without RE*: (*p-value*)	With RE, all predictors (*p-value*)	Without RE, all predictors (*p-value*)
Gender	3.54 *(<0.0001)*	4.26 *(<0.0001)*	3.59 *(<0.0001)*
Age, ordinal	1.04 *(0.3202)*	1.06 *(0.3065)*	1.05 *(0.2790)*
Ethnic group			
Igbo	0.369 *(0.0370)*	0.309 *(0.0551)*	0.382 *(0.0500)*
Igede	0.860 *(0.7414)*	0.888 *(0.8283)*	0.862 *(0.7528)*
Tiv	1.72 *(0.6404)*	1.05 *(0.9747)*	1.03 *(0.9789)*
Hematocrit	0.980 *(0.4084)*	0.979 *(0.4835)*	0.982 *(0.4868)*
Oxygen sat.	0.987 *(0.2141)*	0.983 *(0.1882)*	0.986 *(0.1913)*
Heart rate	0.998 *(0.7070)*	1.00 *(0.5390)*	1.00 *(0.5965)*
Tea-colored urine	0.727 *(0.6093)*	0.511 *(0.3683)*	0.540 *(0.3521)*
Scleral icterus	1.97 *(0.0395)*	2.26 *(0.0563)*	2.12 *(0.0351)*

* RE is the random effect of family.

Note: The backward elimination results are not shown in this table.

The odds of being G6PD deficient were 3.6 times higher in males than in females (p<0.0001). The odds of being G6PD deficient were 0.38 times as high in Igbo children compared to Yoruba children (p = 0.0500). The odds for Igede and Tiv children were not significantly different from Yoruba children (p = 0.7528 and 0.9789 respectively). The odds of being G6PD deficient were 2.1 times higher in children with scleral icterus than those without (p = 0.0351). The magnitude of the scleral icterus effect appeared to be lower if these children also reported tea-colored urine, but the number of children reporting both conditions (N = 10) is too small to be certain.

**Figure 4:**
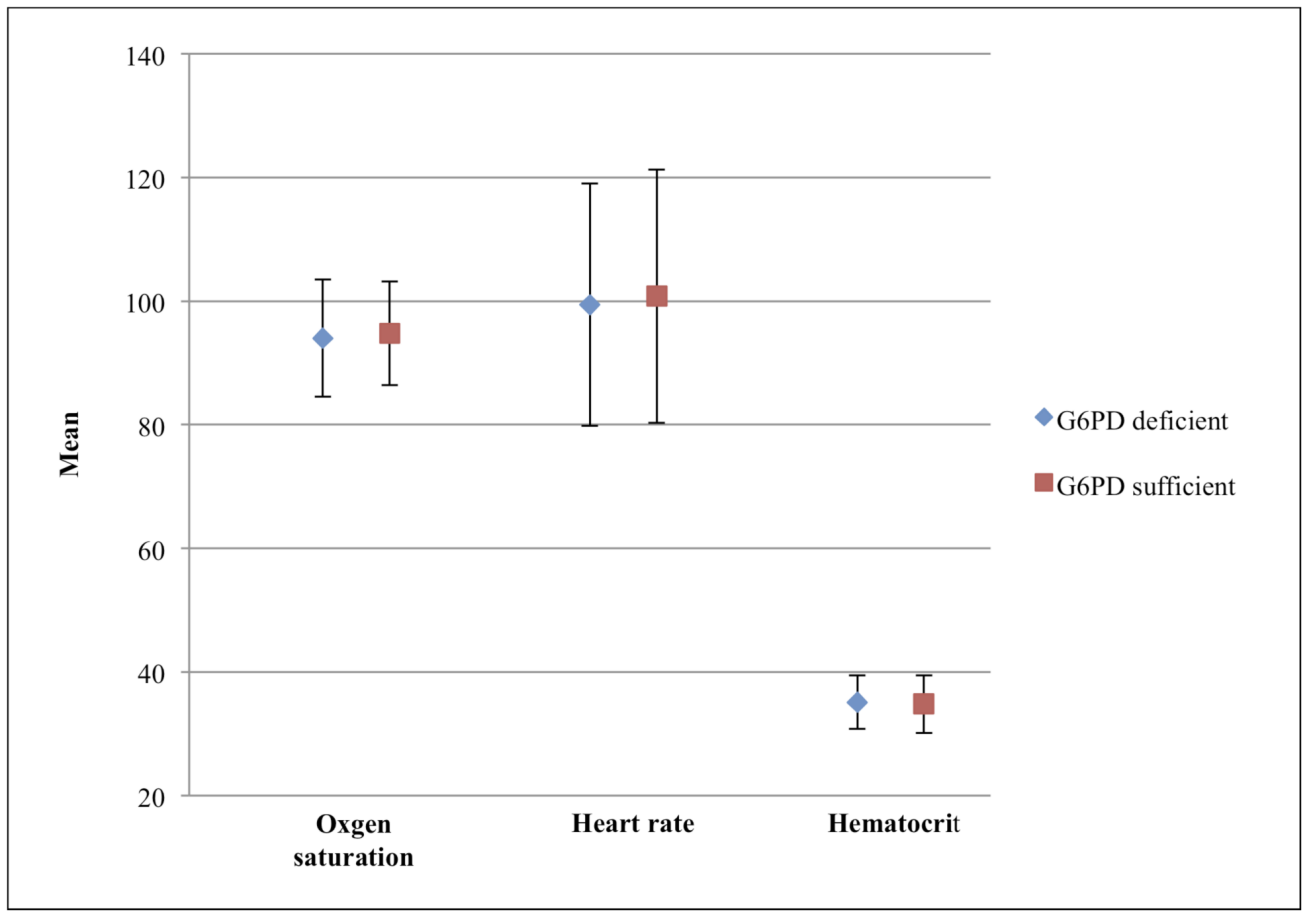
Mean oxygen saturation, heart rate and hematocrit in G6PD deficient and G6PD sufficient children. The Satterthwaite *t-* test was used to compare G6PD deficient to G6PD sufficient. The p-values for oxygen saturation, heart rate and hematocrit were 0.2812, 0.3837, and 0.3515 respectively.

Analysis of oxygen saturation revealed no significant differences between the groups, with a mean saturation of 94.0±9.5% in deficient individuals and 94.8±8.4% in sufficient individuals (p = 0.2812). The rate of hypoxia, defined as an oxygen saturation of less than 90%, did not differ significantly between the two groups at 16.5% in G6PD deficient children and 13.4% in G6PD sufficient individuals (p = 0.2921). Likewise, mean heart rates between the two groups were comparable with a mean of 99.4±19.6 in deficient individuals and 100.8±20.5 in sufficient individuals as shown in Figure 4 (p = 0.3837).

The mean hematocrit of G6PD deficient individuals (35.1±4.3) was similar to that of G6PD sufficient individuals (34.8±4.7, p = 0.3515). The rate of anemia, defined a hematocrit less than or equal to 21, was equally low in both groups: 0.58% in deficient children and 1.8% in sufficient children, (p = 0.9971).

In evaluation of our questions related to hemolysis, the overall rates of jaundice at birth (as reported by the parents), scleral icterus and tea-colored urine were 6.4%, 8.9% and 3.8% respectively. In the univariate analysis G6PD deficient individuals were not more likely than G6PD sufficient individuals to have a history of jaundice at birth (p = 1.000). Among G6PD deficient children, the rates of scleral icterus and tea-colored urine did not vary significantly with age (Table 4, p = 0.9325 and 0.9863 respectively). For scleral icterus and tea-colored urine, unlike all the other predictors, the magnitude of the predicted odds ratio changed slightly in going from the univariate model to the full model (Table 3), indicating that the effect of one may depend on the presence of the other. Having tea-colored urine reduced the odds of being G6PD deficient but the effect was not statistically significant (Table 3, p = 0.3521). The odds of being G6PD deficient were 2.1 times higher in children with scleral icterus than those without (Table 3, p = 0.0351), although the association was not significant after accounting for multiple testing. The magnitude of the scleral icterus effect appeared to be lower if these children also reported tea-colored urine, but the number of children reporting both conditions (N = 10) was too small to be certain.

**Table 4:** Rates of scleral icterus and tea-colored urine among G6PD deficient children of different ages.

	≤6 months	7 months – 2 years	3–6 years	7–10 years	11–15 years	
Scleral icterus % (N†)	0% (1)	16.7% (6)	30% (10)	11.9% (59)	17.2% (29)	p = 0.9325
Tea-colored urine % (N†)	0% (1)	0% (6)	0% (10)	3.4% (59)	3.4% (29)	p = 0.9863

† Total number of G6PD deficient children within age group.

## Discussion

### Summary of Results

The overall prevalence of G6PD deficiency in this study was within the range of what is reported for Nigeria as a whole and is similar to the rate reported in a recent study conducted in the same region of Nigeria[2], [4]. G6PD deficiency is an X-linked condition and as expected, we found that the odds of deficiency were 3.6 times higher in males compared to females.

In this study, we found that ethnic group may be associated with G6PD deficiency, although the effect was not statistically significant after accounting for multiple testing, possibly due to the relatively small number of non-Yoruba children in this study. Yoruba children had the highest prevalence (16.9%) of G6PD deficiency followed by Igede children (10.5%) and children of Igbo (10.1%) and Tiv (5.0%) ethnicity. Igbo children had 0.38 times the odds of being G6PD deficient compared to Yoruba children. The odds for Igede and Tiv children were not significantly different from Yoruba children. To our knowledge, this study is the first to report such differences in prevalence among a seemingly homogenous population. To try to understand these differences, we must review what is currently known about the epidemiology of G6PD deficiency.

The notion that malaria protection has played a role in the global distribution of G6PD deficiency is now widely accepted. Several studies have shown that G6PD deficiency protects against severe malarial anemia although the data is unclear about whether this protection is afforded to only heterozygotes or to homozygotes and hemizygotes as well[1]. *Plasmodium falciparum* is endemic in Nigeria. Therefore, although Yoruba children originate from the southwest of the country and Tiv, Igede and Igbo children from the southeast, they have the same malaria exposure. A selective advantage against malaria explains the high prevalence of G6PD deficiency in Nigeria as a whole, but does not account for the differences in prevalence between the ethnic groups.

Malaria protection is also the reason sickle cell disease disproportionately affects people of African heritage. The genes responsible for sickle cell disease and G6PD deficiency are on different chromosomes and research has shown that the incidence of G6PD deficiency in sickle cell disease is not greater than would be expected by chance [5]. The bulk of evidence also suggests that G6PD deficiency does not increase the incidence of acute anemic episodes or the severity of hemolysis in people with sickle cell disease [6].

In tropical Africa, the G6PD A- variant is thought to account for 90% of G6PD deficiency [1], with about 90% of G6PD A- resulting from the G6PD^202A,376G^ allele [1]. A recent study conducted in The Gambia, West Africa, found that another genotype, G6PD^968C,376G^, was actually the most common cause of G6PD A- in that part of the continent [7], suggesting regional genotypic differences in G6PD deficiency in Africa. The differences in the prevalence of G6PD deficiency seen in this study suggest that there may be more allelic heterogeneity in West Africa than previously thought. This heterogeneity may be both among countries and between ethnic groups in the same country and may lead to different phenotypic manifestations of G6PD deficiency in various sub-populations, as we have found. Genetic mutant verification would delineate this hypothesis.

In this study, we found a slightly lower overall prevalence of G6PD deficiency in females compared to other studies conducted in the same region of Nigeria[4], [8]. G6PD deficient females can be homozygotes or heterozygotes. Heterozygotes can have varying levels of G6PD activity due to X-chromosome inactivation, causing erythrocytic mosaicism[1]. One drawback of the fluorescent spot test is that it only identifies individuals with less than 20% G6PD activity[9]. This can lead to the misclassification of female heterozygotes who are genetically G6PD deficient but have a level of G6PD activity that is marginally greater than 20%. This, as well as the inclusion of Tiv, Igede and Igbo children (who seem to have a lower prevalence of G6PD deficiency) in our sample population, may have contributed to the lower overall prevalence of G6PD deficiency in females in this study compared to other studies.

We found that oxygen saturation and heart rate did not differ significantly between G6PD deficient and sufficient individuals, rendering these measurements unhelpful as screening parameters for differentiating between these two populations. The lack of difference in vital signs is no doubt because hemolysis in G6PD deficiency is largely episodic and triggered by exposure to oxidant stress. The comparable hematocrits between G6PD deficient and sufficient individuals in our sample supports the finding that clinically evident chronic hemolysis is a relatively rare feature of African G6PD deficiency[8]. The unusually high rate of hypoxia among both deficient and sufficient individuals in this study could possibly be an artifact related to difficulties in obtaining accurate pulse oximetry readings on children with perspiring hands (typically <6 years). There is also the possibility that some children with lower oxygen saturations had undiagnosed heart disease, which is prevalent in the developing world.

In this study we found that children with self-reported (or parent-reported) scleral icterus had increased odds of being G6PD deficient, although the association was not significant after accounting for multiple testing, possibly due to the relatively small numbers of children reporting scleral icterus (N = 57). The magnitude of the scleral icterus effect appeared to be lower if these children also reported tea-colored urine, but the number of children reporting both conditions (N = 10) was too small to be certain. Having tea-colored urine reduced the odds of being G6PD deficient but the effect was not statistically significant. This unexpected potential association between tea-colored urine and G6PD deficiency is likely a result of the small overall number of children who reported having tea-colored urine (N = 26) and may reflect recall bias as well as lower than anticipated attention to urine color among children who are old enough to toilet independently.

### Strengths and Limitations

Use of the fluorescent spot test yielded G6PD deficiency prevalence rates that are within the range of what is reported for Nigeria as a whole. The fluorescent spot test is simple and cheap and has a sensitivity of 100% in homozygotes and hemizygotes[10]. The one disadvantage of the test is that it only identifies individuals with less than 20% G6PD activity. This can yield false normal results in female heterozygotes and in deficient individuals who have experienced a recent episode of hemolysis due to the elevated G6PD activity in reticulocytes[9]. Along the same lines, the spot test is not well-suited for screening of neonates due to the presence of reticulocytosis in this population. Other available screening tests are the spectrophotometric and cytochemical assays. The spectrophotometric assay relies on the same principles as the fluorescent spot test and yields false normal results in the same populations. It also requires more training and equipment than the spot test. In the cytochemical assay, G6PD activity causes staining of individual erythrocytes by converting a water-soluble colorless compound into its insoluble dark-colored form through the production of NADPH. This test has a sensitivity of 85% in heterozygotes (compared to 32% and 11% for the fluorescent spot test and spectrophotometric assay respectively)[10]. The major disadvantages of this test are that is it time-consuming, technically difficult and considerably more expensive than the fluorescent spot test.

In this study we screened a large number of Nigerian children for G6PD deficiency. Efforts to obtain a representative sample of participants from different ethnic groups and age groups were limited by the region of the country in which the study was conducted and the number of children who were present and willing to participate in the study on the particular days we visited their school, church or village. This resulted in some ethnic groups and age groups being underrepresented and had a particular impact on the prevalence of G6PD deficiency reported in Tiv children. Sampling a larger population of Tiv children could be the focus of future studies.

We allowed siblings to participate in this study and corrected for any possible within-family correlation in the final multivariate analysis. Children in this study came from different socio-economic backgrounds (their parents were physicians, religious leaders, laboratory technicians, elementary and secondary school teachers and farmers).

One limitation of this study is that we restricted questions about hemolysis to simple questions that we felt a child of at least 8 years of age would be able to answer. This precluded us from asking about confounding or correlated factors such as details of exposure to pro-oxidants and the presence of sickle cell disease or previous episodes of malaria. Even with carefully selected questions, self-reported data is subject to recall bias. This bias as well as the small number of respondents to questions about jaundice at birth prevented us from exploring the relationship between this predictor and G6PD deficiency. Recall bias may also have contributed to the low overall number of children in this study who reported tea-colored urine.

### Conclusions

The high prevalence of G6PD deficiency in Sub-Saharan Africa presents a significant public health burden. Screening for G6PD deficiency has yet to become routine practice in many parts of Nigeria, partly due to cost and a paucity of guidelines regarding which high risk groups should be preferentially screened when general population screening is not possible.

In this study we were able to determine the prevalence of G6PD deficiency in Nigerian sub-populations and found the fluorescent spot test to be a simple and reliable screening tool. The spot test costs less than 3 U.S. cents per person, making it cost-effective for use in the developing world. The one drawback of this test is that it can yield false normal results in female heterozygotes, neonates and deficient individuals who have experienced a recent episode of hemolysis. At this time, the fluorescent spot test is the most viable option for G6PD deficiency screening in Sub-Saharan Africa, but its limitations highlight the need for continued research into cheap and reliable screening methods.

The possible association between scleral icterus and G6PD deficiency discovered in this study, if confirmed in larger studies, could be used by those designing screening programs to identify children who are more likely to be G6PD deficient and should be prioritized for screening. Further research needs to be done, however, to better delineate the relationship between G6PD deficiency and other causes of hemolysis such as sickle cell disease and malaria.

The differences in the prevalence of G6PD deficiency between the ethnic groups in this study suggests that our understanding of the epidemiologic and genetic factors that contribute to G6PD deficiency in Sub-Saharan Africa is still incomplete. As reliable screening tests and molecular diagnostic methods become more widely available we will gain a better understanding of the origins of disease phenotype and new information about how G6PD deficiency has spread over the African continent with the movement of populations.
